# Al_2_O_3_-MgO Supported Ni, Mo, and NiMo Mixed Phosphidic-Sulphidic Phase for Hydrotreating of Stearic and Oleic Acids Into Green Diesel

**DOI:** 10.3389/fchem.2022.880051

**Published:** 2022-05-09

**Authors:** Esneyder Puello-Polo, Dana Arias, Edgar Márquez

**Affiliations:** ^1^ Grupo de Investigación en Oxi/Hidrotratamiento Catalítico y Nuevos Materiales, Programa de Química-Ciencias Básicas Universidad del Atlántico, Barranquilla, Colombia; ^2^ Grupo de Investigación en Química y Biología, Departamento de Química y Biología, Universidad del Norte, Barranquilla, Colombia

**Keywords:** hydrotreating, Ni-Mo phosphidic-sulphidic, fatty acids, bifunctional sites, pseudo-first-order rate constants, adsorption constants

## Abstract

The effect of the sulfur and metal-type content of MoP-S/*γ*-Al_2_O_3_-MgO, NiMoP-S/*γ*-Al_2_O_3_-MgO, and NiP-S/*γ*-Al_2_O_3_-MgO phosphide on hydroprocessing (HDO, HDCx-HDCn, HCK, HYD, and HYG) of fatty acids was studied. The catalysts were characterized by XRF, XRD, textural properties, XPS, Raman, Py-TPD, and EDS elemental mapping. The chemical analyses by X-ray fluorescence (XRF), EDS elemental mapping, and CHNS-O elemental analysis showed stoichiometric values Al/Mg = 38–40, Mo:Ni:P ∼ 1, and S ≤ 4.5 wt % (this value means that the molar ratio Mo:S ∼ 1.0:1.6, i.e., MoS_2_); also EDS elemental mapping confirmed the presence of Mo, Ni, Al, O, P, Mg, and S with good distribution on Al_2_O_3_-MgO. The impregnation of metals leads to a decrease in the surface area and pore volume as follows NiMoP-S/*γ*-Al_2_O_3_-MgO < MoP-S/*γ*-Al_2_O_3_-MgO < NiP-S/*γ*-Al_2_O_3_-MgO < Al_2_O_3_-MgO < Al_2_O_3_ (unimodal pore size distribution), propitiating a pseudo bimodal pore size distribution with Dp-BJH between ∼5–7 nm and 11.8–14.2 nm for the presence of MgO. XRD confirmed differences between metallic phosphates and phosphides, and XPS confirmed the presence at the surface of Mo^δ+^(0 < δ+ < 2), Mo^4+^, Mo^6+^, Ni^δ+^(0 < δ+ < 2), Ni^2+^, S^2−^, SO_4_^2−^, P^δ+^, and P^5+^ species. Raman revealed the presence of MoS_2_ only in MoP-S/*γ*-Al_2_O_3_-MgO and NiMoP-S/*γ*-Al_2_O_3_-MgO, while the NiMoP-S/γ-Al_2_O_3_-MgO catalyst had a more significant number of Brønsted and Lewis sites, although the increasing temperature decreased the Lewis sites. MoP-S/*γ*-Al_2_O_3_-MgO was more active at HDO showing the highest production rate for octadecane of 53 mol/(g_cat_·h), whereas NiP-S/*γ*-Al_2_O_3_-MgO was more active at HDCx-HDCn [45 mol/(g_cat_·h)] and HCK [6 mol/(g_cat_·h)]; meanwhile, NiMoP-S/*γ*-Al_2_O_3_-MgO had a mix of HDO [47 mol/(g_cat_·h)] and HDCx-HDCn [41 mol/(g_cat_·h)]. This showed production towards octadecane, heptadecane, and light hydrocarbons, meaning that the fatty acids were deoxygenated through bifunctional sites for hydrogenation (HYD) and hydrogenolysis (HYG) as follows: MoP-S/*γ*-Al_2_O_3_-MgO (K_1_ = 0.08 and K_2_ = 0.03 L/mol) < NiMoP-S/*γ*-Al_2_O_3_-MgO (K_1_ = 0.25 and K_2_ = 0.45 L/mol) < NiP-S/*γ*-Al_2_O_3_-MgO (K_1_ = 2.5 and K_2_ = 6.5 L/mol). For this reason, we considered that phosphide acts as a structural promoter with sulfur on its surface as a “mixed phosphidic-sulphidic species”, allowing the largest generation of heptadecane and octadecane by the presence of BRIM sites for HYD and CUS sites for HYG.

## GRAPHICAL ABSTRACT

The sulfur and metal-type content in MoP-S/*γ*-Al_2_O_3_-MgO, NiMoP-S/*γ*-Al_2_O_3_-MgO, and NiP-S/*γ*-Al_2_O_3_-MgO influence the reaction pathways for hydrotreating fatty acids, improving the dispersion of MoS_2_ on phosphide “mixed phosphidic-sulphidic species” and increasing the number of Lewis and Brönsted sites, which correlates well with the HDO and HDCx-HDCn pathways.
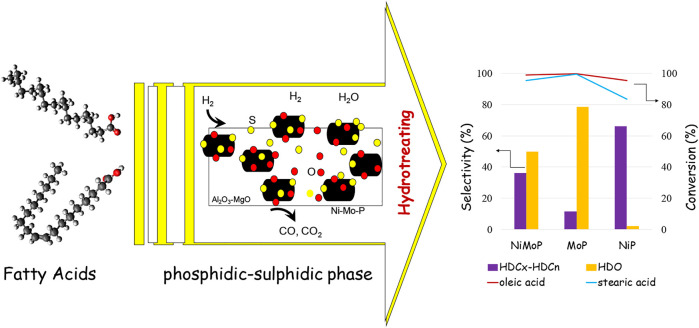

## Highlights

• The phosphide acts as a structural promoter with sulfur on its surface, improving the catalytic activity.
• The Ni-Mo-P-S catalysts upgrade fatty acids through HDO using two bifunctional sites (hydrogenation and hydrogenolysis).
• Catalysts presented an Ni-Mo mixed phosphidic-sulphidic phase that could be the active phase in HDO on presulfiding phosphide catalysts.
• Bifunctional catalysts promote selection of fatty acid HYD by HDO and HDCx-HDCn pathways.
• Kinetics allowed describe the mechanism of oleic and stearic acids HYD by a Ni-Mo mixed phosphidic-sulphidic phase.

## Introduction

Fossil fuels are the primary source of energy worldwide; however, the high demand to ensure the global economic and social development model has increasingly limited their availability. Thus, the increased consumption of oil derivatives quickly reduces world crude oil reserves (Gousi et al., 2017). Hence, there is concern about conserving non-renewable natural resources and evaluating alternative energies that gradually replace natural gas, coal, and fossil fuels in generation and not in the transport sector (Pearlson et al., 2013; Patil and Vaidya, 2018). Therefore, it seems essential to have more technically and economically viable possibilities to replace petroleum-derived fuels in the short and medium term. In this respect, biodiesel (FAME) is an alternative. However, FAME presents a series of disadvantages such as high oxygen content (35–40% w/w), high cloud point temperature, and poor oxidation stability which restrict its further development, corrosion in the equipment caused by the acid–base and salt-containing wastewater, a large number of neutralizing and purification steps in its manufacture, high viscosity which influences the increase of NOx emissions, and that the used catalyst cannot be recycled, which increases the capital and operating costs (Souza Macedo et al., 2019; Bezergianni and Dimitriadis, 2013).

A fact that has been gaining attention in recent years is the use of biofuels that have compositions resembling that of fossil-derived fuel and that avoid the presence of glycerol (petro-diesel), which is called “renewable diesel” (Donnis et al., 2009; Wang et al., 2018). This renewable diesel is composed mainly of an n-paraffin long chain that could ultimately replace petroleum-derived diesel fuel because both share identical composition and energy densities that are obtained from renewable sources (such as vegetable oils or animal fats) using catalytic hydrotreatment (HDT), and can be co-processed together with the heavy crude oil of equivalent molecular weights in conventional oil refineries (Yusuf et al., 2011; Helwani et al., 2009).

In general, the hydrotreating of triglycerides and fatty acids is the removal of oxygen yielding high-quality diesel fuel by three reaction pathways: decarboxylation (DCx), decarbonylation (DCn), and hydrodeoxygenation (HDO) (Gosselink et al., 2013; Susanto et al., 2016). Three central catalyst systems have been applied to the hydrotreatment/deoxygenation of triglycerides and fatty acids as follows: 1) Ni(Co)-Mo sulfides; 2) noble metals, mainly including Pt or Pd-based catalysts; and 3) non-noble transition metals (e.g., Ni, Mo, and others) (Immer et al., 2010; Sotelo-Boyás et al., 2011; Duan, 2017). However, sulfide catalysts are easily deactivated by oxidation (decreasing activity and contamination of products due to sulfur leaching), and noble metals are expensive; hence, these deficiencies restrict their practical use. On the other hand, non-noble transition metal catalysts have high catalytic activity and low cost, they are applicable in the deoxygenation process (Arend et al., 2011; Cecilia et al., 2013; Kandel et al., 2014; Smirnov et al., 2016), although they are poisoned by the nitrogenous compounds present in the fatty acids used for increased stability (Kamal-Eldin and Yanishlieva, 2002). In this regard, the transition metals: carbides, nitrides, borides, and phosphides-based solids have been identified as potential catalysts due to their attractive hydrogen transfer properties similar to those of noble metals. At the same time, they are sulfur, nitrogen, and oxygen-tolerant and have high thermal stability (leaching resistance), which is due to their particular structure resulting from the ligand and ensemble effects of carbon, nitrogen, boron, and phosphorus (Wang et al., 2011; Bui et al., 2012; Han et al., 2012).

The nature of the support can influence the product distribution of the HDO reaction (Kubička and Kaluža, 2010). The most widely used hydrotreating (HDT) support is alumina, which has strong interactions with compounds containing two oxygen atoms, showing deactivation due to coke formation (Centeno et al., 1995). In particular, Centeno et al. (1995) demonstrated that an essential cause for catalyst deactivation using alumina as the support in HDO of guaiacol, catechol, phenol, 4-methyl acetophenone, and p-cresol was due to coke formation. Hence, optimal acidity formulations have been proposed because they confer high catalyst hydrocracking (HCK) activity with a low coke deposition rate (Leyva et al., 2007). Notably, the addition of some oxides containing basic sites to the conventional support of hydrotreating catalysts, such as magnesium oxide (MgO) (Solis et al., 2004; Klicpera and Zdražil, 2002), decreases the metal-support interaction that promotes high dispersion on the catalyst support, cracking with low olefins hydrogenation, and the basic character of the support could inhibit coke formation (Klicpera and Zdražil, 2002). However, pure MgO is less convenient for co-hydrotreating of oxygen-containing feed because water and COx are formed during HDO which causes textural instability due to the presence of water that can destroy part of the surface of MgO (Kaluža et al., 2019). However, Yang et al., using CoMo(P)/MgO in HDO of bio-crude, highlighted that MgO-supported catalysts increased yields of reduced hydrocarbon products, such as benzene and cyclohexyl-aromatics, that is, less hydrocracking (Yang et al., 2009).

In addition, phosphorus was found to show beneficial effects on the activity of the hydrotreating reaction due to decreased coke formation and formation of new Lewis and Bronsted acid sites on the catalyst surface (Ferdous et al., 2004). Recently, Zhang et al. (2016) tested the addition of phosphorus to sulfided CoMoP/Al_2_O_3_ for HDO of pyrolysis oil, reducing the oxygen content from 41.8 wt % to 3 wt .%, which suggests that phosphorus promotes the activation of hydrogen in the hydrotreatment process. Hence, transition metal phosphide catalysts have been studied extensively because they are economical and less susceptible to hydrogenolysis of the C-C bond (Berenguer et al., 2016; Gonçalves et al., 2017). However, for triglycerides, HDO and fatty acids require two catalytically active sites: 1) a hydrogenation metallic site and 2) acid site for hydrogenolysis, dehydration, and isomerization (Zhao et al., 2011; Yu et al., 2019). Regarding those, it has been shown that transition metal phosphides are bifunctional in nature by the metal and P-OH groups/unreduced Ni^2+^, respectively (Infantes-Molina et al., 2015; Lee and Oyama, 2006).

There are many reports of transition metal phosphides (especially Ni_2_P) because they have shown excellent HDO performance (Lee and Oyama, 2006; Wang et al., 2002), with relevant studies on the active phase in HDO; hence, Z. Zhang et al. considering the works of Sawhill et al. (2005) and Li et al. (2014) showed that the Ni_2_P phase is very active in the HDS and dehydrogenation processes; thus they studied the effects of the P/Ni ratio and the Ni content of phosphides on the HDO of methyl laurate to hydrocarbons. The results showed that HDO was promoted by increasing the P/Ni ratio and the Ni content in Ni_3_P, Ni_12_P_5,_ and Ni_2_P. Likewise, Ni_x_P_y_ showed much lower activity for decarbonylation, C-C hydrogenation, and methanation (Zhang et al., 2016).

Yang et al. (2012) performed a comparison of Ni_2_P/SBA-15 and Ni/SBA-15 on HDO of methyl oleate, where Ni_2_P/SBA-15 produced a high content of long-chain hydrocarbons, while Ni/SBA-15, due to the cracking reaction, gave a wider distribution of hydrocarbons. Guan et al. (2016) demonstrated the excellent HDO activities of methyl palmitate using nickel phosphides supported on MCM-41. However, in this study, Ni-based catalysts generally suffered from a high level of coke formation resulting in severe catalytic deactivation (Khzouz et al., 2013). Therefore, another active site needed to be added. Thus, several studies have been carried out with metallic combinations in phosphide catalysts (Feitosa et al., 2016; Chen et al., 2014a).

Rensel et al. (2013) revealed that FeMoP catalysts were highly selective for the rupture of the C-O bond in HDO of aryl ethers and phenol. A. Cho investigated the HDO of 2-MTHF on NiFeP, in which a synergistic effect between Ni and Fe on selectivity was observed (Cho et al., 2012). Pan et al. (2015) suggested that the catalytic performance of NiMoP catalysts in the HDO of methyl laurate is due to the charge transfer of Ni to Mo in NiMoP_2_ or the incorporation of Mo into the structure of Ni_2_P. In addition, Chen et al. found that the increase in TOF correlated with the increase of Mo on silica-supported Ni-Mo phosphides (Chen et al., 2014a; Pan et al., 2015). Yun et al. (2017) synthesized a series of NiMo bimetallic phosphides supported on MCM-41, and the HDO of γ-valerolactone was tested. The activity was related with the proportion of Ni in the catalyst, in the following order: Ni_2_P/MCM-41 > NiMo(3:1)P/MCM-41 > NiMo(1:1)P/MCM-41 ∼ (Ni_2_P + MoP)/MCM-41 > NiMo(1:3)P/MCM-41 > MoP/MCM-41, suggesting that the Ni was the main active site, while Mo determined the selectivity. Although sulfided MgO-supported catalysts have been the object of a limited number of research studies (Zdražil, 2003; Klicpera and Zdražil, 2000); catalytic application of Al_2_O_3_-MgO supported phosphides to HDO is almost nil. Furthermore, many studies hold that transition metal phosphides are potential catalysts in hydrotreating, but the exact nature of the active sites in these systems is still a matter of debate, and it is believed to be a stable phosphosulfide surface at hydrotreating conditions (Nelson et al., 2006). Hence, the results previously shown provide an incentive to explore the comparison of a series of Al_2_O_3_-MgO-supported nickel–molybdenum phosphides modified with sulfur, in which their activity is tested in HDO of fatty acids and gives insight into the catalyst structure-performance relationship.

## Experimental

### Preparation of Alumina Modified With Magnesium

The Al_2_O_3_-MgO catalytic supports with an Al/Mg atomic ratio = 38 were prepared by modifying traditional sol-gel synthesis (Puello-Polo et al., 2021; Méndez et al., 2013). In a typical experiment, appropriate amounts of aluminum (III) isopropylate {Al[OCH(CH_3_)_2_]_3_, 99.8%, Sigma- Aldrich} was dispersed in 50 ml of isopropanol [(CH_3_)_2_CHOH, 98%, Sigma-Aldrich] under magnetic stirring at about 343 K until a homogeneous solution was obtained.

An aqueous solution of magnesium nitrate hexahydrate [6.1 mmol of Mg(NO_3_)_2_·6H_2_O, 98%, Sigma-Aldrich] was added to the solution of Al[OCH(CH_3_)_2_]_3_/(CH_3_)_2_CHOH at 350 K for 4 h and then cooled to room temperature until the polymerized solution of Mg(NO_3_)_2_·6H_2_O/Al[OCH(CH_3_)_2_]_3_ in alcoholic medium was obtained. The solution was then slowly added to a surfactants solution that was obtained by homogenizing 56.5 mmol of tetramethylammonium hydroxide (TMAOH, 25% in H_2_O, Sigma-Aldrich) in 30 ml of deionized water followed by the addition of 18.1 mmol of hexadecyltrimethylammonium bromide (99%, Sigma-Aldrich) until complete dispersion.

Simultaneously, the pH was adjusted between 8 and 10 with ammonium hydroxide (NH_4_OH, 28.0%–30.0% NH_3_ basis, Sigma-Aldrich), and kept under magnetic agitation for 2 h. The resulting mixture was aged for 5 days without stirring, and the formed gel was washed with deionized water until neutral pH. The solid obtained was dried at 393 K for 18 h, then the sample was calcined in a tubular furnace and treated with air at a flow rate of 50 cm^3^ min^−1^ at 823 K for 8 h. Finally, the collected solid sample was milled, and the particles of a 100–140 mesh were collected for use (denoted as Al_2_O_3_-MgO).

### Preparation of Catalyst and Precursors Supported on Al_2_O_3_-MgO

In all the runs, 3.5 g of Al_2_O_3_-MgO (Al/Mg = 38; mesh 100–140) was impregnated in excess of the pore volume to provide a uniform coating surface (Puello-Polo et al., 2021). The supported precursor was prepared by dropwise adding an aqueous solution of nickel (II) nitrate hexahydrate [Ni(NO_3_)_2_·6H_2_O, Aldrich, 99%] and/or ammonium heptamolybdate [(NH_4_)_6_Mo_7_O_24_·4H_2_O, Aldrich, 99%] with ammonium dihydrogen phosphate [(NH_4_)H_2_PO_4_, Aldrich, 99%] in stoichiometric amounts of Mo:Ni:P = 1:1:1 (20 wt.% Mo) to a flask containing the support, under stirring at 343 K. The impregnation step lasted until removal of the solvent by evaporation. Finally, the mass obtained was further dried at 373 K for 12 h and the sample was calcined at 823 K for 4 h. The precursors were transferred to a fixed-bed tubular stainless-steel reactor (30 cm long, 20 mm I.D.) with an axial thermowell containing a thermocouple centered in the catalyst bed deposited on a quartz fiber bed. A tubular electrical furnace controlled by a temperature programmer (Electro Salgado Ltd.) was used to heat the reactor. The amount of the sample was approximately 2 g/batch. Pure H_2_ was passed through the sample at a total flow rate of 50 cm^3^ min^−1^. The temperature was increased at a linear rate of 1°C min^−1^ to the final temperature (973 K), which was held for 1 h (Liu et al., 2017). The samples were quenched to room temperature and then passivated by a 1% air/N_2_ mixture for 1 h.

### Catalyst and Support Characterization

Elemental analysis for Mo, Ni, P, S, and Mg was determined by X-ray fluorescence using a MagixPro PW–2440 Philips instrument. The textural characteristics of the catalysts were determined employing the physisorption of N_2_ at 77 K using a Micromeritics 3FLEX™ instrument. The Brunauer–Emmett–Teller multipoint method (BET) calculated the surface areas of samples, and total pore volume and pore size distribution were determined from the desorption branch of the isotherm using the Barret–Joyner–Halenda (BJH) model (Barrett et al., 1951). The total pore volume was estimated by summation of microporous and mesoporous volumes. The surface area due to micropores (S_micro_) was obtained from a mass balance, i.e., S_micro_ = S_BET_ − S_ext_ (Puello-Polo et al., 2018). XRD analysis of the samples was carried out using a SIEMENS D-5005 diffractometer with a Cu Kα radiation source (λ = 1.5418 Å) and Ni filter, within the range 5° ≤ 2θ ≤ 90°. For the identification of the crystallographic phase(s) (International Centre for Diffraction Data, 1995), the JCPDS data files were preferentially used for (NH_4_)_3_PO_4_(MoO_3_)_12_•4H_2_O (card no. 09-0408), MoP (card no. 24-0771), NiMoP (card no. 031-0873), and γ-Al_2_O_3_ (card no. 10-0425). Raman spectra of the phosphides were obtained in a Horiba Scientific Raman microspectrometer with LabRAM HR Evolution at room temperature using the 532 nm line and a between 41.1 and 56.1 μW He–Ne laser excitation source through an Olympus TM BX41 optical microscope. FTIR measured the pyridine adsorption on the different samples with the aim to monitor both the type (Brønsted or Lewis acidic sites) and their relative abundance (Crépeau et al., 2006). A thin wafer of the pure catalyst powder was made (8 mg cm^−2^) and placed into a special IR cell; then, the sample was immediately pretreated under vacuum at 773 K for 4 h. Afterwards, the sample temperature was decreased to 423 K and exposed to pyridine vapor (1.5 m bar pressure) for 5 min. The physically adsorbed pyridine fraction was removed by degassing the sample for 30 min, and the IR spectra were collected. Spectra were recorded in 256 scans using a Nicolet FTIR spectrometer, and the data analysis was performed with the spectrometer software Omnic 8.1. The adsorbed pyridine on Brönsted and Lewis acid sites was calculated under different desorption temperatures (50, 100, 200, and 300°C). The acidity due to Brönsted and Lewis sites (L and B, respectively), in μmol Py/g, was calculated as:

$$L=\frac{\pi /4\left(D^{2}\right)A_{1450}}{\left(IMEC_{L}\right)m_{solid}}\;B=\frac{\pi /4\left(D^{2}\right)A_{1540}}{\left(IMEC_{B}\right)m_{solid}}$$

Where D is the diameter of self-supported wafers, A1450 and A1540 are the integrated area below the bands at 1,450 and 1,540 cm^−1^ in the absorption spectrum of pyridine, IMECB and IMECL are the integrated molar extinction coefficients corresponding to the bands at 1,450 and 1,540 cm^−1^, IMECL = 222 cm/μmol and IMECB = 167 cm/μmol (Emeis, 1993), and m_solid_ is the mass of the sample (g).

The sulfided phosphides’ surface composition was determined through X-ray photoelectron spectroscopy (XPS) with a Thermo Scientific K-Alpha spectrometer, equipped with a dual (non-monochromatic) Mg/Al anode, operated at 400 W and under a vacuum better than 10^–9^ torr. Calibration of the instrument was carried out employing the Au 4f_7/2_ line at 83.9 eV. A dual flood gun source of low-energy electrons and Ar^+^ ions was used during all measurements to prevent surface charging. The internal referencing of binding energies was made using the dominating Al 2p band of the support at 74.4 eV. After *in situ* sulfiding for XPS analysis, samples were immersed in purified n-hexane and quickly transferred to the XPS instrument, where the solvent was slowly removed under vacuum (Farojr and Dossantos, 2006). Curve fitting of the spectra was carried out using the XPSPEAK version 4.1 and XPS GRAPH programs, employing a standard nonlinear least-squares curve fitting routine and mixed Gaussian/Lorentzian peak shapes of variable proportion, after baseline subtraction by the Shirley method.

Scanning electron microscopy (SEM) with energy-dispersive X-ray spectroscopy (EDX) analysis were undertaken using a field emission scanning electron microscope (JEOL, model JSM-7800F, Japan) operated at 1 kV. The EDX chemical elemental mapping images and elemental analysis were acquired simultaneously at 15 kV using the energy-dispersive X-ray spectroscopy interconnected with the same JEOL 7800F instrument.

Sulfur elemental analysis of the mixed phosphidic-sulphidic phase was carried out using a combustion method using a Fisons EA 1108 CHNS-O analyzer.

### Hydrotreatment Test of Fatty Acids

To remove the passivation layer and attain a stable and reproducible Ni-Mo mixed phosphidic-sulphidic phase (Puello-Polo and Brito, 2008), before the catalytic reaction, the phosphide and passivated forms were activated *in situ* under a 0.5 vol % CS_2_/heptane mixture and H_2_ (50 cm^3^ min^−1^) at 300°C for 1 h. The hydrotreatment of the mix of fatty acids (oleic and stearic acids) was conducted under conventional hydrotreating conditions in a 600 ml batch reactor (Trejo et al., 2005). In a typical reaction, 0.5 g of catalyst, 1.6 g of fatty acids (oleic acid: stearic acid = 1:1), and 78.4 g of n-dodecane were introduced into the batch reactor, and subsequently, the reactor was purged three times by N_2_ at ambient temperature and then pressured to 3.1 MPa H_2_. The reaction mixture was then heated from room temperature to reaction temperature (573 K) and target H_2_ pressure (5 MPa H_2_) for 6 h under constant stirring (300 rpm). The consumption during the reaction of fatty acids and the products contained in the liquid phase were collected and injected into a CG-2014 Shimadzu equipped with a flame ionization detector (FID). The products were identified and confirmed by GC-MS Agilent technology 7890B GC System-5977A MSD and compared to standards. The effluents sampling of the reactor occurred at 0, 90, 180, 270, and 360 min, and the catalytic activities of the catalysts were determined (Puello-Polo et al., 2021). The conversion of fatty acids was calculated as the proportion of fatty acids converted concerning the fatty acids feedstock at 6 h of reaction as defined in Eq. 1:

$$\%\;FA\;conversion=\frac{FA_{0}-FA_{t}}{FA_{0}}\times 100$$ (1)

where FAo and FAt are the fatty acid molar concentration (mol L^−1^) at the beginning and at 6 h, respectively.

Additionally, the yield of fatty acids HYD was calculated as the proportion of a product formed in relation to the fatty acids consumed during the reaction as defined in Eq. 2:

$$\text{\%}\;\text{yield}\;i=\frac{{\text{C}}_{\text{i}}}{{\text{FA}}_{0}}\times 100$$ (2)

where FAo and Ci are the fatty acid molar concentration (mol L^−1^) at the beginning and product formed at 6 h, respectively.

Both the supporting alumina and the Mg-modified alumina showed negligible fatty acids conversion in the reaction. Established procedures verified the absence of mass and heat flow transport effects (Froment et al., 2011). All experiments reported in this work (synthesis protocols, characterizations, and catalytic activity measurements) were carried out at least in triplicate. Good reproducibility was verified, better than 10% in all quantitative measurements.

**SCHEME 1:**
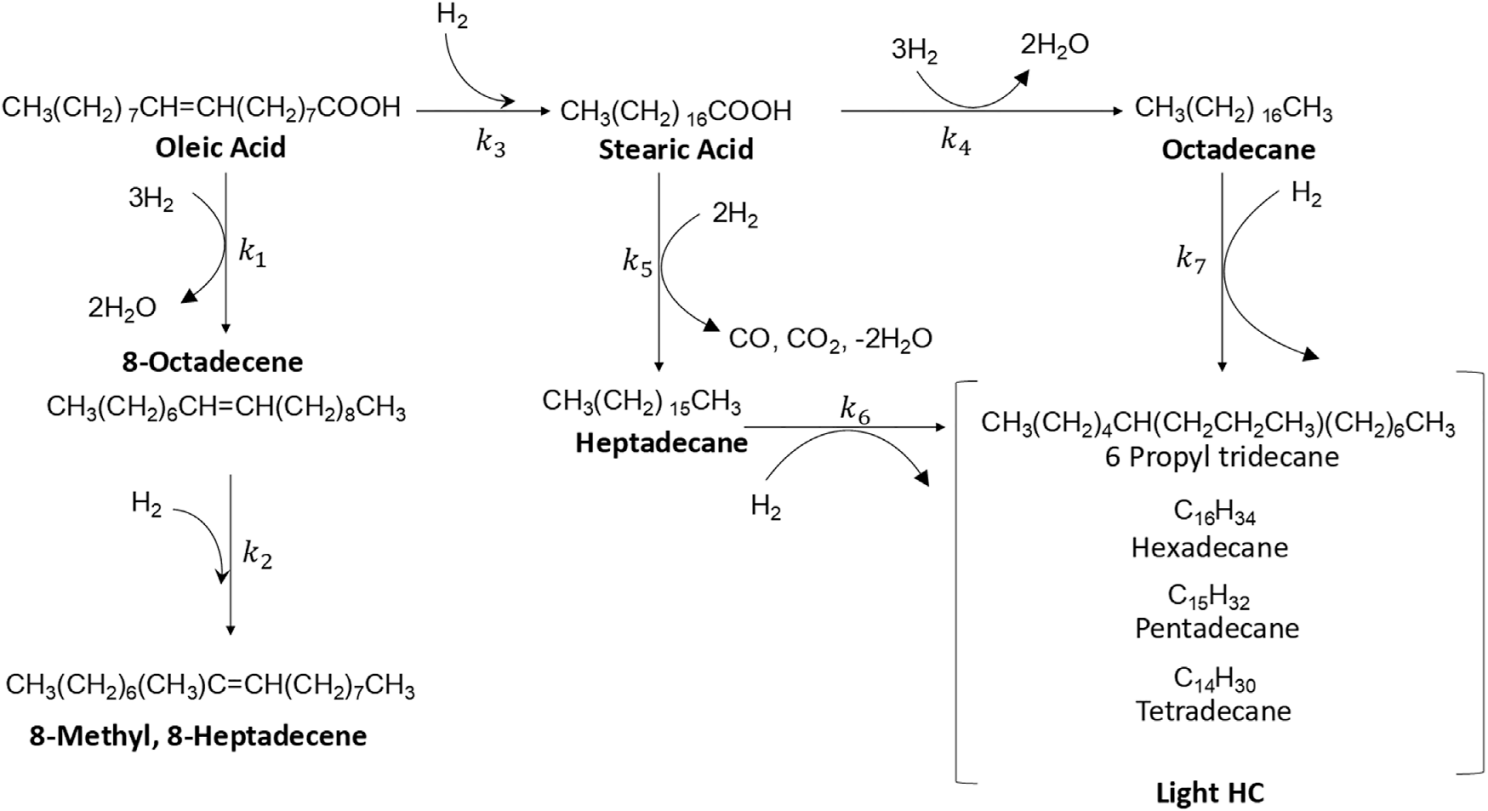
Reaction pathways for hydrotreating oleic and stearic acids.

All the reactions were assumed irreversible due to the excess of hydrogen (considered constant), whereby the reactions were considered pseudo-first-order. The semi-empirical kinetic model of fatty acids HYD is the generalized Hinshelwood-Hougen-Watson model (Satterfield et al., 1980). It was calculated according to the results obtained by Kumar and Hachemi, suggesting a modified HYD reaction network as presented in Scheme 1 (Hachemi and Murzin, 2018; Kumar et al., 2014), the reaction rate was defined in Eq. 3.

$$r_{ij}=\frac{k_{ij}C_{i}}{{\left(1+\sum_{m}K_{m}C_{m}\right)}^{Z}}$$ (3)

Where rij (mol/L h) is the rate of conversion of compound i to compound j, C (mol/L) is the concentration, kij (L/mol h) is the apparent kinetic rate constant (including surface reaction and adsorption contributions), and Km (L/mol) is the adsorption parameter of individual compounds. Thus, Z represents the number of surface sites required for the reaction.

In Scheme 1, a variety of complex reactions can be seen during the one-step hydroprocessing of fatty acids, including isomerization, hydrodeoxygenation, hydrocracking, hydrodecarbonylation, and hydrodecarboxylation. This reveals that oleic acid (unsaturated acids) undergoes hydrosaturation to form stearic acid and isomerization to produce 8-octadecene and 8-methyl-8-heptadecene; subsequently, the saturated stearic acid is converted into long-chain hydrocarbons by dehydration, decarbonylation, and decarboxylation reactions to form heptadecane, octadecane, and light hydrocarbons (light HC, <C17) as identified by GC-MS, which accounted for more than 98% of the total products.

The system of differential equations to calculate the apparent rate and equilibrium adsorption constants was solved using the Mathematics-Wolfram software version 11.1. The nonlinear parameter estimations were calculated until converged by minimizing the deviation from theoretical and experimental concentrations (objective function) at different times using the Levenberg–Marquardt algorithm of GNU Octave version 4.2.2 through the Optim package, with the constraint of non-negativity. In this approach, the apparent rate and equilibrium adsorption constants of the oleic and stearic acids HYD were calculated as k_1_, k_2_, k_3_, k_4_, k_5_, k_6_, and k_7_ and K_1_ and K_2_, respectively.

Finally, the fatty acids HYD activities of the catalysts were reported as pseudo-first-order rate constant for oleic and stearic acid disappearance normalized by the moles of oleic or stearic acids per weight (m_cat_, g) of the catalyst after 6 h of reaction time. Therefore, the HDO, DCx-DCn, and hydrocracking (HCK) activities were reported after 6 h of reaction time as defined in Eqs 4–6:

$$\text{H}\text{D}\text{O}\;\text{a}\text{c}\text{t}\text{i}\text{v}\text{i}\text{t}\text{y}=k_{1}+k_{4}$$ (4)

$$\text{D}\text{C}\text{x}-\text{D}\text{C}\text{n}\;\text{a}\text{c}\text{t}\text{i}\text{v}\text{i}\text{t}\text{y}=k_{5}$$ (5)

$$\text{H}\text{C}\text{K}\;\text{a}\text{c}\text{t}\text{i}\text{v}\text{i}\text{t}\text{y}=k_{6}+k_{7}$$ (6)

## Results and Discussion

### Chemical Analysis

Results obtained from XRF and EDX chemical analyses of Al_2_O_3_-MgO, NiP-S/*γ*-Al_2_O_3_-MgO, MoP-S/*γ*-Al_2_O_3_-MgO, and NiMoP-S/*γ*-Al_2_O_3_-MgO are shown in Table 1. The differences between experimental and nominal contents for all catalysts were within experimental accuracy, whose deviations could be related to the steps applied to synthesizing the supports and catalysts (Puello-Polo et al., 2020). Hence, the Al/Mg, Ni:Mo, Ni:P, and Mo:Ni:P atomic ratios proposed were similar to the designed formulation of the supports and catalysts in this work. On the other hand, sulfur elemental analysis of the mixed phosphidic-sulphidic phase showed low content of sulfur <4 wt %, suggesting that the phosphides were not degraded because the total generation of metallic sulfides with our catalysts resulted in 10–23 wt % of S (see CHONS chemical analyses); therefore, only changes in the surface were observed as will be shown in the XPS analysis (Nelson et al., 2006).

**TABLE 1:** Experimental composition of the Al_2_O_3_-MgO supported NiMo mixed phosphidic-sulphidic phase.

Solid	Experimental composition (wt%)-XRF					Experimental composition (wt%)-EDS					CHONS elemental analysis
	Mo	Ni	P	S	$\frac{\text{Al}}{Mg}$	Mo	Ni	P	S	$\frac{\text{Al}}{Mg}$	% wt S
NiP-S/*γ*-Al_2_O_3_-MgO		11.6	6.9	2.2	40		8.1	5.5	1.0	41	2.0
MoP-S/*γ*-Al_2_O_3_-MgO	19.0		6.1	6.2	40	17.5		4.9	2.1	37	2.9
NiMoP-S/*γ*-Al_2_O_3_-MgO	20.1	10.5	6.2	5.1	38	21.5	8.5	7.1	3.3	41	4.5

wt %: percentage by weight; composition nominal: Ni:Mo:P = 1.0, Al/Mg = 38 (2 wt % MgO); γ-Al_2_O_3_-MgO experimental data: Al/Mg = 42 (3.5 wt % MgO).

### XRD Analysis

**FIGURE 1:**
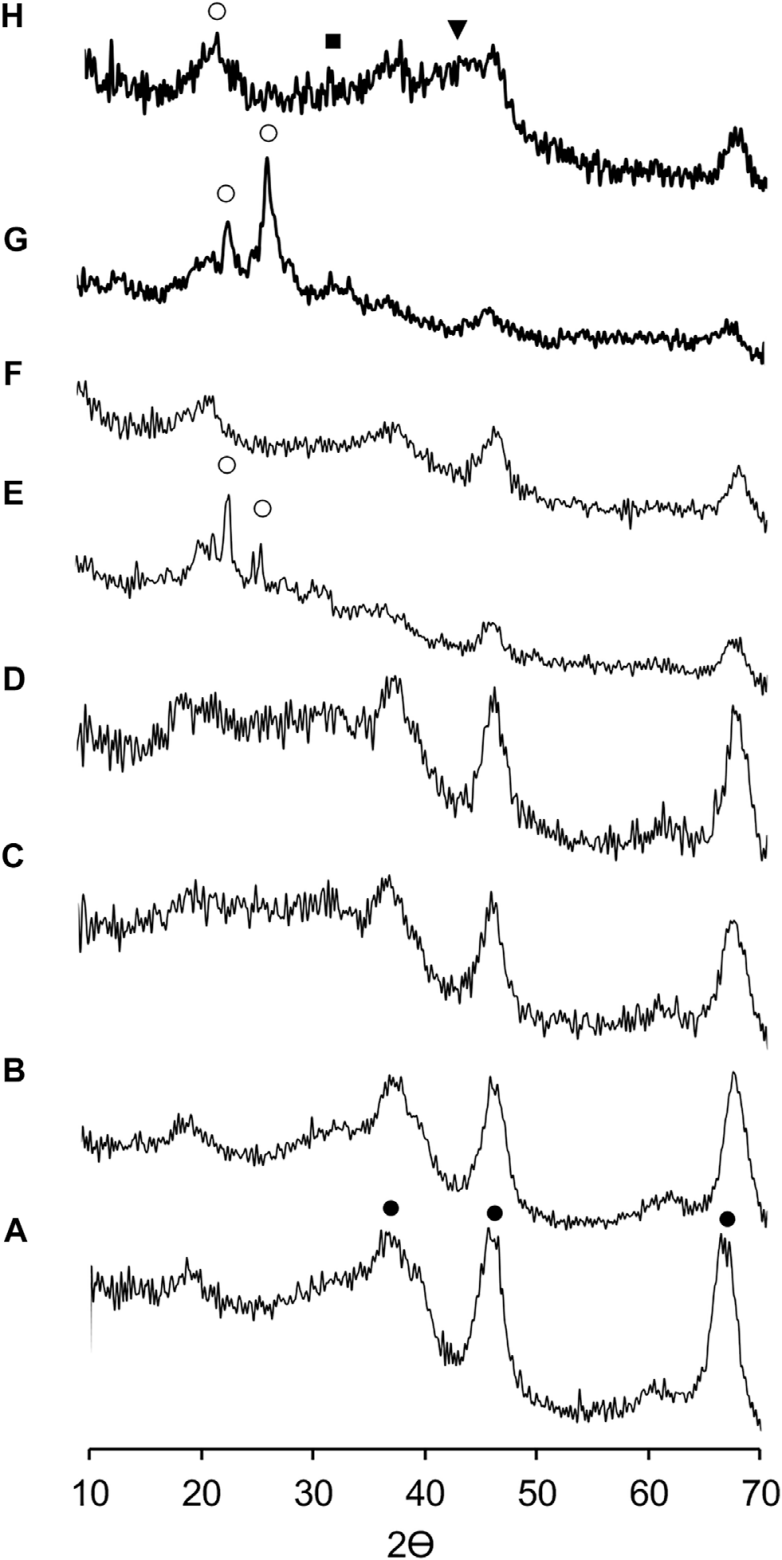
X-ray diffraction patterns of supports and the Al_2_O_3_-MgO supported NiMo mixed phosphidic-sulphidic phase: **(A)** Al_2_O_3_, **(B)** Al_2_O_3_-MgO, **(C)** NiP-O/*γ*-Al_2_O_3_-MgO, **(D)** NiP-S/*γ*-Al_2_O_3_-MgO, **(E)** MoP-O/*γ*-Al_2_O_3_-MgO, **(F)** MoP-S/*γ*-Al_2_O_3_-MgO, **(G)** NiMoP-O/*γ*-Al_2_O_3_-MgO, and **(H)** NiMoP-S/*γ*-Al_2_O_3_-MgO. (•) *γ*-Al_2_O_3_, (■) MoP, (▼) NiMoP, and (○) (NH_4_)_3_PO_4_(MoO_3_)_12_•4H_2_O.

The Figure 1 shows the XRD patterns of mixed phosphidic-sulphidic phase and their precursors (metallic phosphates). The XRD patterns revealed differences between metallic phosphates and mixed phosphidic-sulphidic phase except for the NiP-O/γ-Al_2_O_3_-MgO (C and D patterns), which does not show characteristic signs of a metal phosphate or phosphide. It is worth mentioning that it is difficult to distinguish the diffraction peaks, referring to phosphide/sulfide and MgO (∼2 wt%) in all the catalysts, probably because alumina amorphism, crystallites size (too small to give XRD signals) or the particles of phosphidic-sulphidic composite well dispersed on the γ-Al_2_O_3_-MgO matrix. However, due to the changes in the difractograms between metallic phosphates and phosphides, it lets assume that under the reaction conditions it is likely to generate these types of species, such as MoP at 2θ = 32.17 and γ-Al_2_O_3_ at 2θ = 67.10, 45.90, 37.64 in MoP-S/γ-Al_2_O_3_-MgO (E and F patterns); and NiMoP at 2θ = 43.19; (NH_4_)_3_PO_4_(MoO_3_)_12_•4H_2_O at 2θ = 26.47, 21.51 and γ-Al_2_O_3_ for NiMoP-S/γ-Al_2_O_3_-MgO as will be displayed in XPS (G and H patterns).

### Raman

**FIGURE 2:**
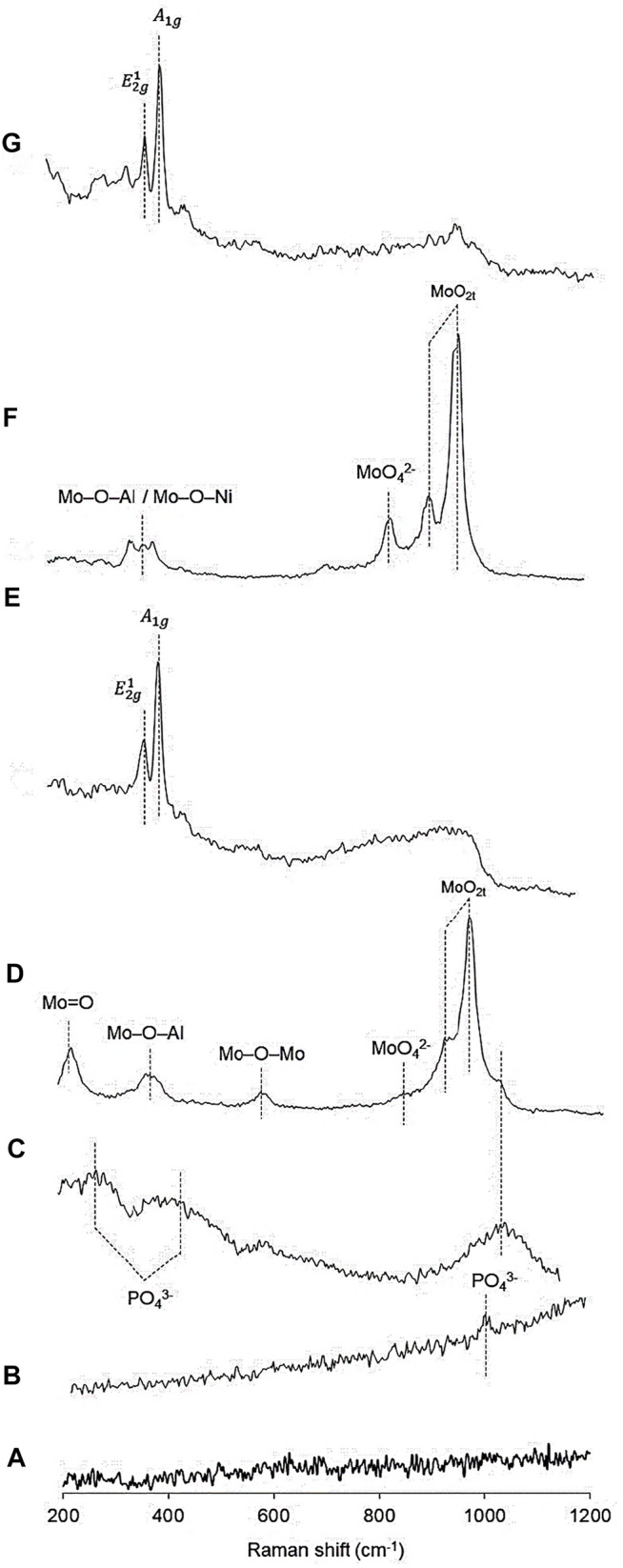
Raman spectra of the Al_2_O_3_-MgO supported NiMo mixed phosphidic-sulphidic phase**: (A)** Al_2_O_3_-MgO, **(B)** NiP-O/*γ*-Al_2_O_3_-MgO, **(C)** NiP-S/*γ*-Al_2_O_3_-MgO, **(D)** MoP-O/*γ*-Al_2_O_3_-MgO, **(E)** MoP-S/*γ*-Al_2_O_3_-MgO, **(F)** NiMoP-O/*γ*-Al_2_O_3_-MgO, and **(G)** NiMoP-S/*γ*-Al_2_O_3_-MgO.

Figure 2 shows the Raman spectra of the precursors and catalysts which revealed the absence of bands corresponding to the *γ*-Al_2_O_3_-MgO support. The NiP-O/*γ*-Al_2_O_3_-MgO precursor presented the main band at approximately 1,000 cm^−1^, characteristic of antisymmetric stretching modes of P-O bonds (PO_4_^3−^ stretching) (Butt et al., 2004; Yang et al., 2019), while NiP-S/*γ*-Al_2_O_3_-MgO showed three peaks around 425, 300, and 1,126 cm^−1^ that were assigned to the PO_4_^3−^ bending modes (Yang et al., 2019). MoP-O/*γ*-Al_2_O_3_-MgO showed bands at 1,018 (shoulder), 960, 922, 830 (shoulder), 588, 382, and 226 cm^−1^ (shoulder) that could be assigned to the PO_3_^4−^ group, MoO_2t_ symmetric stretching, MoO_4_^2−^, Mo–O–Mo bridge stretching, stretching of Mo–O–Al entities, and terminal Mo=O of octahedral MoO_4_^2−^ species, respectively (Wang et al., 2016; Botto et al., 1992). NiMoP-O/*γ*-Al_2_O_3_-MgO displayed bands at 1,000 (shoulder), 952, 906, 838, 360–390, and 224 cm^−1^ that could be assigned to the PO_3_^4−^ group, MoO_2t_ symmetric stretching, MoO_4_^2−^, stretching of Mo–O–Al or Mo–O–Ni entities, and terminal Mo=O of octahedral MoO_4_^2−^ species, respectively (Botto et al., 1992). The Raman spectra of the Mo and NiMo phosphides after passivation and sulfidation at 573 K are shown in Figure 2, which do not present Raman bands attributed to phosphates and oxidic phases (E and G). In this figure, MoP-S/*γ*-Al_2_O_3_-MgO and NiMoP-S/*γ*-Al_2_O_3_-MgO presented two typical Raman peaks at 380 and 405 cm^−1^ for the E^1^_2g_ and A_1g_ active modes, characteristic of the bulk or multilayered MoS_2_ (Li et al., 2012). We suppose that the phosphides phase was absent because of the processes of oxidation-sulfiding or the metal/P ratio (Yang et al., 2020). The bands’ intensity and shape depended on the solid obtained; thus, the vibration bands’ intensity corresponding to the mixed phosphidic-sulphidic phase was higher than phosphates due to hydrogenation treatment, which is likely to induce the growth of crystallites.

### Textural Properties

**FIGURE 3:**
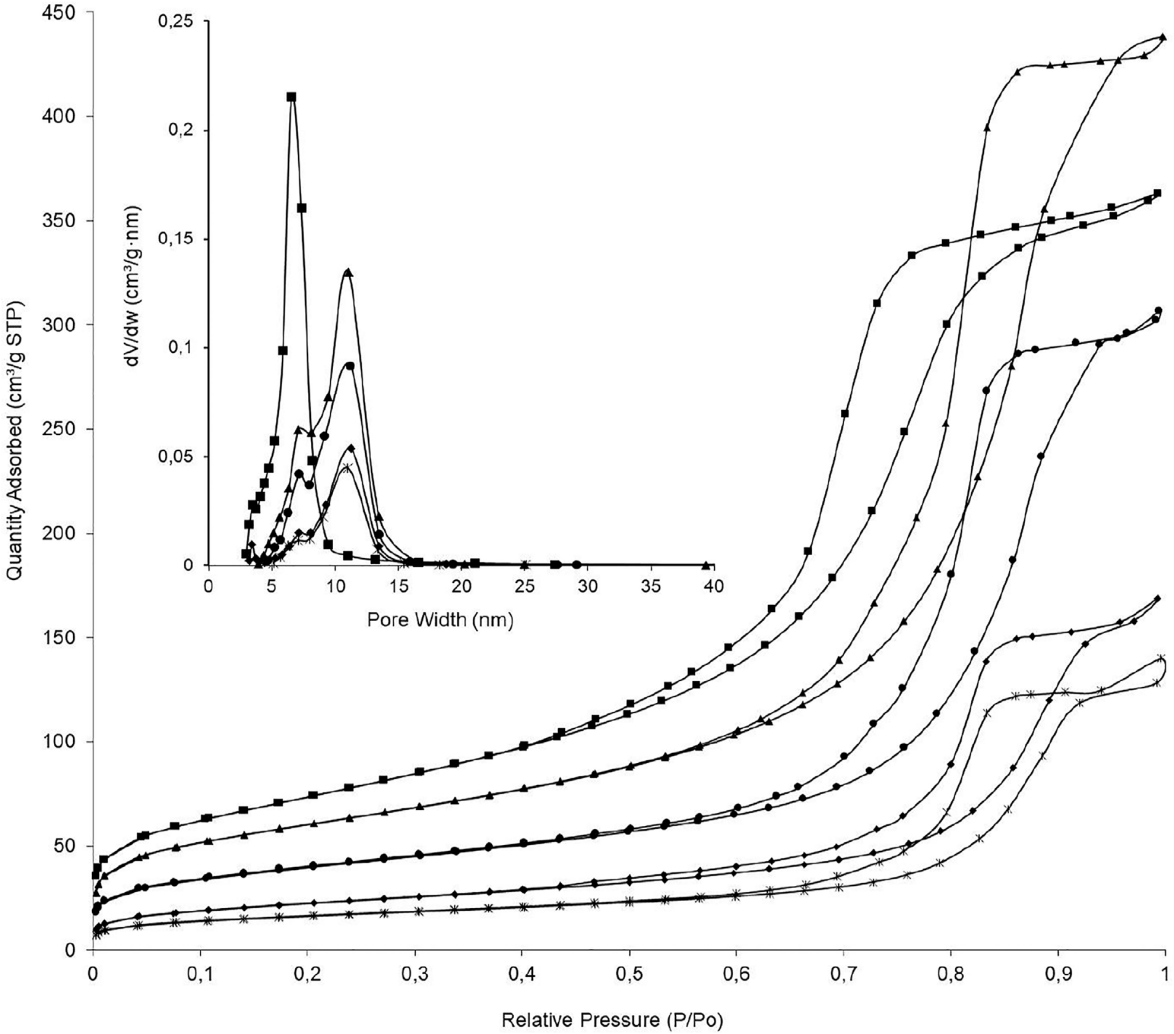
Adsorption/desorption isotherms of N_2_ at 77 K and pore size distribution of the supports and the Al_2_O_3_-MgO supported NiMo mixed phosphidic-sulphidic phase. (■) Al_2_O_3_, (▲) Al_2_O_3_-MgO, (•) NiP-S/*γ*-Al_2_O_3_-MgO, (♦) MoP-S/*γ*-Al_2_O_3_-MgO, and (ж) NiMoP-S/*γ*-Al_2_O_3_-MgO.

All the N_2_ physisorption isotherms of Al_2_O_3_, Al_2_O_3_-MgO, NiP-S/*γ*-Al_2_O_3_-MgO, MoP-S/*γ*-Al_2_O_3_-MgO, and NiMoP-S/*γ*-Al_2_O_3_-MgO were type IV in the IUPAC classification (Figure 3) (Thommes et al., 2015). The hysteresis loops showed that Al_2_O_3_, Al_2_O_3_-MgO, and NiP-S/*γ*-Al_2_O_3_-MgO were H1 due to uniform mesopores; while MoP-S/*γ*-Al_2_O_3_-MgO and NiMoP-S/*γ*-Al_2_O_3_-MgO were H2 related with ink-bottle and uniform mesopores. The isotherms behavior revealed the composite type’s influence on textural properties of the catalysts. The surface area and pore volume of all solids were shown to increase as follows except for Al_2_O_3_ and Al_2_O_3_-MgO: NiMoP-S/*γ*-Al_2_O_3_-MgO < MoP-S/*γ*-Al_2_O_3_-MgO < NiP-S/*γ*-Al_2_O_3_-MgO < Al_2_O_3_-MgO < Al_2_O_3_. This behavior is related to the Ni, Mo, P, and Mg migration into the support pores, generating microporosity during the impregnation process, as shown in Table 2 for the S_micro_/S_BET_ ratio, although larger pores were of interest in this study due to the large size of the fatty acids.

**TABLE 2:** Textural properties of the Al_2_O_3_-MgO supported NiMo mixed phosphidic-sulphidic phase.

Solid	Specific surface area and porous characteristics							
	S_BET_ (m^2^/g)	V_meso_ (cm^3^/g)	V_micro_ (cm^3^/g)	V_T_ (cm^3^/g)	D_p_ (nm)	S_EXT_	S_micro_	S_micro_/S_T_
*γ*-Al_2_O_3_	265	0.566	0.0006	0.57	6.5	258	7	2.6
*γ*-Al_2_O_3_-MgO	213	0.659	0.008	0.67	8.1; 11.1	193	20	9.4
NiP-S/*γ*-Al_2_O_3_-MgO	141	0.471	0.005	0.48	7.3; 11.2	129	12	8.5
MoP-S/*γ*-Al_2_O_3_-MgO	80	0.258	0.0005	0.26	7.0; 11.2	77	3	3.8
NiMoP-S/*γ*-Al_2_O_3_-MgO	58	0.193	0.0014	0.19	7.0; 11.0	54	4	6.9

S_BET_, BET surface area; S_micro_, micropores surface area; S_ext_, external surface area; S_micro_/S_BET_, fraction of the micropores area to the total surface area; V_T_, total volume of pores; Dp, mean pore diameter; V_meso_, mesopores volume; V_micro_, micropores volume.

The Al_2_O_3_-MgO support increased the pore volume and diameter, which means that the Mg^2+^ ions were incorporated into the Al_2_O_3_ structure [r Mg^2+^(0.072 nm) > r Al^3+^ (0.051 nm)] due to the sol-gel method that forms Al-O-Mg bonding in the process of gelation (Puello-Polo et al., 2018; Ueno et al., 1983). In this respect, it is expected that when Mg is incorporated into the alumina structure it would improve the dispersion of Ni, Mo, and P on the support.

The pore diameters of the catalysts and supports were principally located in the mesoporous range (2–50 nm) (Thommes et al., 2015), with values of BJH mesopore mean size between 6.5 and 11.2 nm in the order: Al_2_O_3_ < NiMoP-S/*γ*-Al_2_O_3_-MgO < MoP-S/*γ*-Al_2_O_3_-MgO < NiP-S/*γ*-Al_2_O_3_-MgO < Al_2_O_3_-MgO. In addition, the Al_2_O_3_-MgO and phosphides isotherms showed a slight jump in their adsorption branch at high relative pressures, suggesting a certain bimodality in pore size distribution with pore sizes below ∼7 and 11 nm, whereas Al_2_O_3_ displayed a unimodal pore size distribution (see Table 2 and the box in Figure 3) (Uhlig et al., 2018).

### XPS Analysis

**FIGURE 4:**
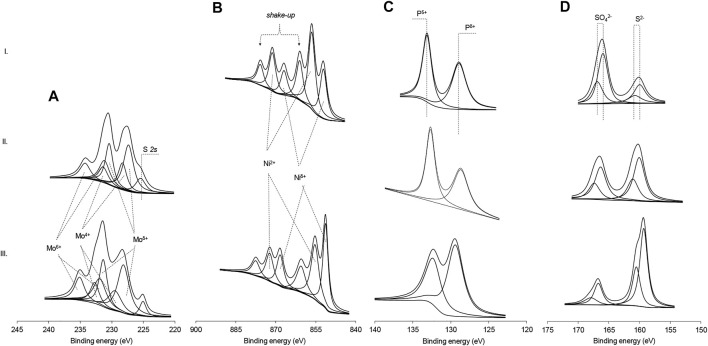
X-ray photoelectron spectra of Mo 3d, Ni 2p, P 2p, and S 2p regions of the Al_2_O_3_-MgO-supported NiMo mixed phosphidic-sulphidic phase: **(A)** Mo 3d, **(B)** Ni 2p, **(C)** P 2p, and **(D)** S 2p. (I) NiP-S/*γ*-Al_2_O_3_-MgO; (II) MoP-S/*γ*-Al_2_O_3_-MgO; and (III) NiMoP-S/*γ*-Al_2_O_3_-MgO.

The XPS technique was applied to find out the composition and show that metal phosphides were formed in NiP-S/*γ*-Al_2_O_3_-MgO, MoP-S/*γ*-Al_2_O_3_-MgO, and NiMoP-S/*γ*-Al_2_O_3_-MgO. In the Ni 2p region spectra of NiP-S/*γ*-Al_2_O_3_-MgO, Figure 4B shows three Ni 2p_3/2_ peaks centered at 852.4, 856, and 862 eV (Hsu and Lin, 2017). These signals are attributed to Ni^δ+^ (0 < δ+ < 2) of nickel phosphide, Ni^2+^ ions interacting with phosphate or sulfates, and the strong shake-up lines characteristic of Ni^2+^ species in an Ni-O-P/Ni-O-S matrix, respectively. Note that the electron binding energy of Ni^δ+^ was more extensive than that of metallic Ni, suggesting the charge transfer from Ni to P (Gonçalves et al., 2017). Likewise, Figure 4A shows the Mo 3d_5/2-3/2_ region spectra of MoP-S/*γ*-Al_2_O_3_-MgO and NiMoP-S/*γ*-Al_2_O_3_-MgO catalysts. The peaks confirm the presence on the surface of Mo^δ+^(0 < δ+ < 2; Mo 3d_5/2_ = 228.7eV), Mo^4+^ (Mo 3d_5/2_ = 229 eV), and Mo^6+^ (Mo 3d_5/2_ = 232.5 eV) species due to Mo-P, MoS_2,_ and MoO_3_, respectively (Phillips et al., 2002). It can be observed that the presence of a promoter is reflected in a higher amount of Mo^δ+^ and Mo^4+^ species (sulfided). Additionally, in the Mo 3d_5/2-3/2_ region, a shoulder at 226.5 eV was found, corresponding to the 2 s signal of sulfur, which can be confirmed by the presence of two bands in the S 2p_3/2_ region (Weber et al., 1996): a sign at 161.7 eV due to terminal disulfide and/or sulfide (S^2−^) ligands in MoS_2_ or NiMoS phases and the signal at 168.9 eV, which can be assigned to SO_4_^2−^ (Figure 4D). The signals due to sulfur species and its proportion are much more important for MoP-S/*γ*-Al_2_O_3_-MgO and NiMoP-S/*γ*-Al_2_O_3_-MgO, whereas that partial S substitution of P in NiP-S/*γ*-Al_2_O_3_-MgO was less as shown by Raman results. Figure 4C shows the XPS spectra in the P 2p region, revealing two kinds of phosphorus signals with different relative contents on all catalysts. The peaks at 129.3 and 134.2 eV correspond to the reduced P species in the phosphides and the PO_4_^3−^ species that were probably derived from the surface passivation (Sawhill et al., 2005). The binding energy of reduced P species is smaller than the element P, indicating that there is a charge transfer from Ni or Mo to P.

Curve fitting of the spectra in the P 2p region revealed that the amount of PO_4_^3−^ species was much less than that of the reduced P (Table 3). This further demonstrates that more P atoms interacted with Ni or Mo atoms. Hence, the relative quantities of low oxidation state Mo, Ni, P, and S species was higher in NiMoP-S/*γ*-Al_2_O_3_-MgO, suggesting a synergistic effect between the metals by the enhanced charge transfer from metals to P.

**TABLE 3:** Atomic chemical composition of the Mo 3d, Ni 2p, P 2p, and S 2p regions in the Al_2_O_3_-MgO supported NiMo mixed phosphidic-sulphidic phase.

Catalyst	Mo 3d_5/2_-3d_3/2_			Ni 2p_3/2_-2p_1/2_		S 2p_3/2_-2p_1/2_		P 2p_3/2_-2p_1/2_	
	Mo^δ+^ 228.7 eV (at %)	Mo^4+^ 229 eV (at %)	Mo^6+^ 232.5 eV (at %)	Ni^δ+^ 852.4 eV (at %)	Ni^2+^ 856 eV (at %)	S^2−^ 161.7 eV (at %)	SO_4_^2−^168.9 eV (at %)	P^δ+^ 129.4 eV (at %)	P^5+^ 133.6 eV (at %)
NiP-S/*γ*-Al_2_O_3_-MgO				0.78	1.21	0.43	0.33	3.47	3.19
MoP-S/*γ*-Al_2_O_3_-MgO	1.72	0.82	1.02			0.50	0.26	3.45	3.21
NiMoP-S/*γ*-Al_2_O_3_-MgO	1.91	0.80	0.84	1.01	0.98	0.58	0.18	4.04	2.62

### FTIR of Adsorbed Pyridine

**FIGURE 5:**
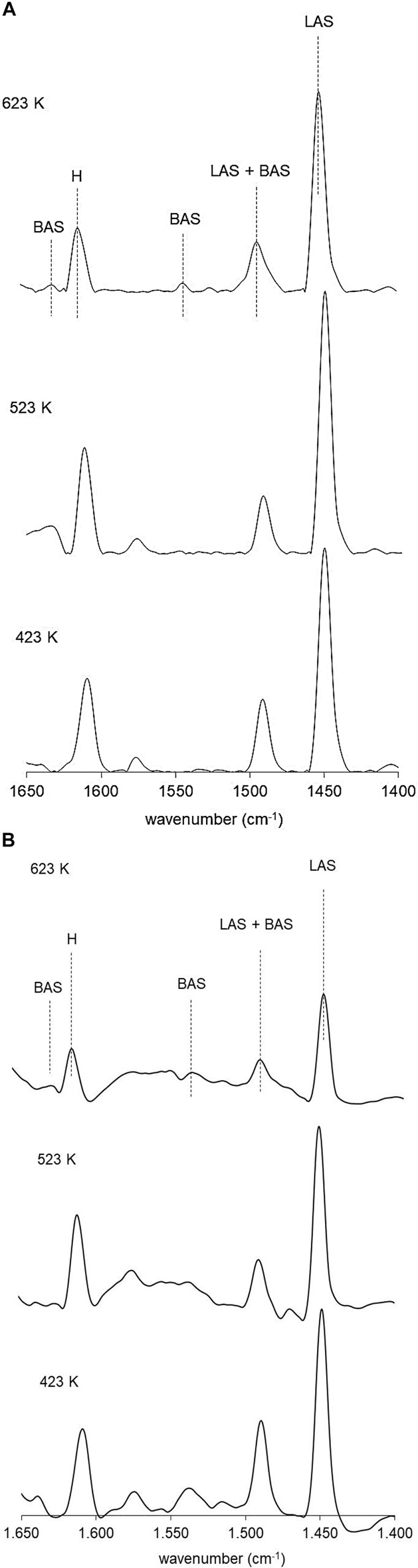
**(A)** FTIR spectra of pyridine adsorption over the NiP-S/*γ*-Al_2_O_3_-MgO catalyst. **(B)** FTIR spectra of pyridine adsorption over the NiMoP-S/*γ*-Al_2_O_3_-MgO catalyst.

Figures 5A,B present the infrared spectra in the region between 1,700 and 1,400 cm^−1^ of the pyridine vibration bands vs. thermodesorption temperature for NiP-S/γ-Al_2_O_3_-MgO and NiMoP-S/γ-Al_2_O_3_-MgO activated at 423, 523, and 623 K. This method was used to identify the types and strength of acid sites in the different materials (Crépeau et al., 2006). Figure 5 shows bands at 1,450, 1,540/1,638, 1,490, and 1,608 cm^−1^, attributed to pyridine bound to Lewis acid sites, Brönsted acid sites, the sum of Brönsted and Lewis acid sites, and hydrogen bonding with Al_2_O_3_ surface hydroxyl groups, respectively (Barzetti et al., 1996). This figure shows that the Lewis acid is dominant over the Brönsted acid for all the catalysts, as reported by Oyama (Lee and Oyama, 2006). The NiMoP-S/γ-Al_2_O_3_-MgO catalyst has a more significant number of Brønsted and Lewis sites as follows: 423 K (BS = 34.2 μmol/g and LS = 106.6 μmol/g), 523 K (BS = 15.2 μmol/g and LS = 89.8 μmol/g), and 623 K (BS = 15.7 μmol/g and LS = 36.9 μmol/g). However, as the temperature increases, it is observed that the amount of the Lewis sites in NiMoP-S/γ-Al_2_O_3_-MgO was much less than that of NiP-S/γ-Al_2_O_3_-MgO [423 K (BS = 1.4 μmol/g and LS = 94.7 μmol/g), 523 K (BS = 1.0 μmol/g and LS = 61.9 μmol/g), and 623 K (BS = 0.9 μmol/g and LS = 57.7 μmol/g)], suggesting that these sites in the NiP-S/γ-Al_2_O_3_-MgO catalyst are stronger that NiMoP-S/γ-Al_2_O_3_-MgO; moreover, their Brönsted acid sites did not produce appreciable changes (Ledesma et al., 2020). On the other hand, the presence of Mo increased the content of Brönsted acid sites; then it is possible to presume that the MoP-S/γ-Al_2_O_3_-MgO catalyst will have a more significant number of Brönsted acid sites than NiP-S/γ-Al_2_O_3_-MgO.

### SEM Analysis With Energy Dispersive X-Ray Spectroscopy and Elemental Mapping

**FIGURE 6:**
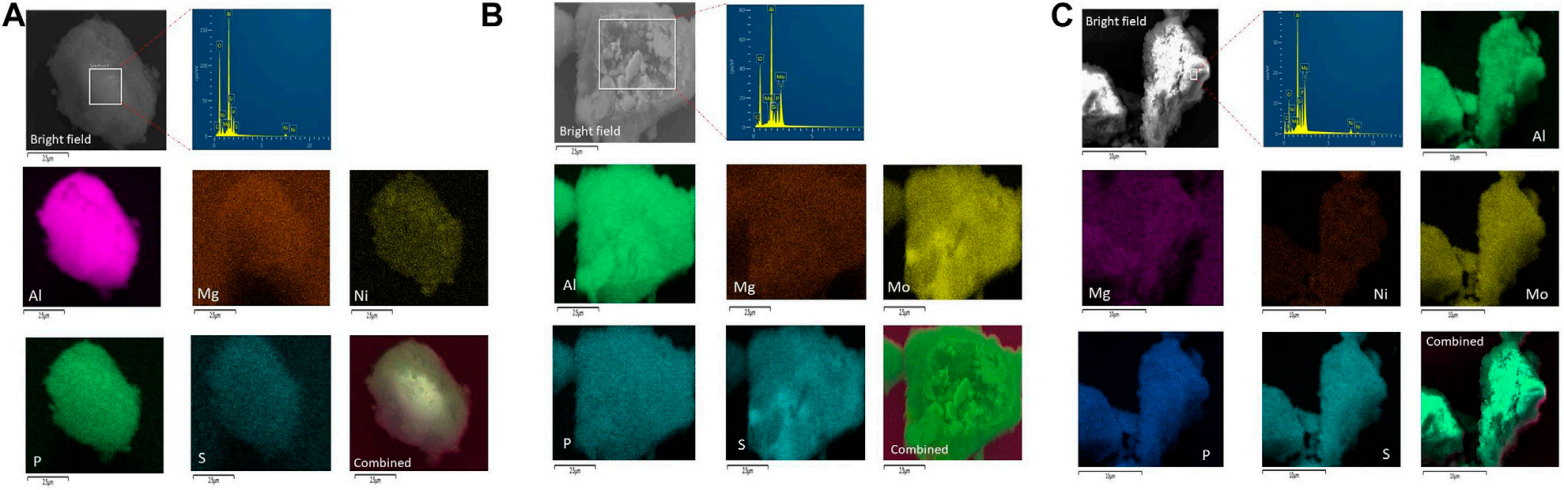
**(A)** EDX elemental mapping of Ni, Al, O, P, Mg, and S over the NiP-S/*γ*-Al_2_O_3_-MgO catalyst. **(B)** EDX elemental mapping of Mo, Ni, Al, O, P, Mg, and S over the MoP-S/*γ*-Al_2_O_3_-MgO catalyst. **(C)** EDX elemental mapping of Mo, Ni, Al, O, P, Mg, and S over the NiMoP-S/*γ*-Al_2_O_3_-MgO catalyst.

Figures 6B,C exhibit the SEM microscopy of the MoP-S/γ-Al_2_O_3_-MgO and NiMoP-S/γ-Al_2_O_3_-MgO catalysts, which showed morphologies consisting of particle cumulus with irregular geometries, except for NiP-S/γ-Al_2_O_3_-MgO (Figure 6A), which presented a morphology regular with particle sizes smaller than NiMoP-S/γ-Al_2_O_3_-MgO and MoP-S/γ-Al_2_O_3_-MgO. The EDS elemental mapping confirmed the well-distributed presence of the atoms constituting the catalysts on the support, i.e., Mo, Ni, Al, O, P, Mg, and S. However, the micrographs show the greater intensity of mapping in a few catalyst zones for Ni and Mo atoms associated to the solubility of the precursors, suggesting that some of these atoms could precipitate. But they are not dispersing on the whole selected area forming a metal rich-aggregate “cluster” in the NiP-S/γ-Al_2_O_3_-MgO and MoP-S/γ-Al_2_O_3_-MgO catalysts (Puello-Polo et al., 2020).

### Catalytic Tests

**FIGURE 7:**
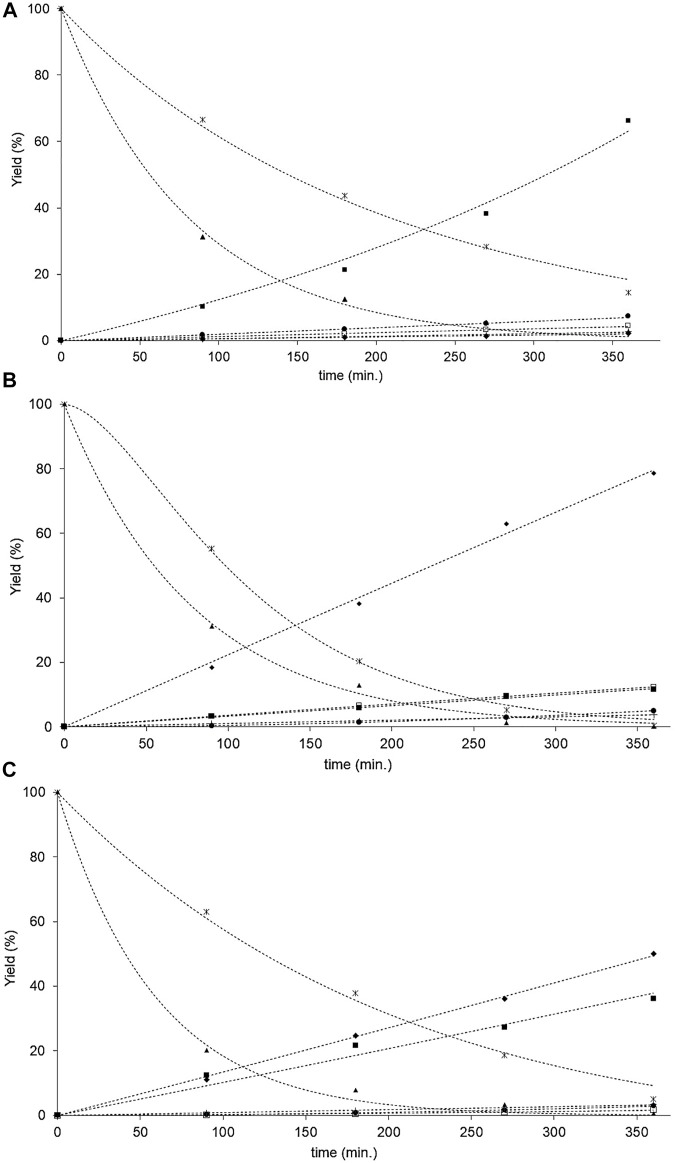
**(A)** The oleic and stearic acids HYD activities in function of reaction time over the NiP-S/*γ*-Al_2_O_3_-MgO catalyst. (▲) Oleic acid, (ж) stearic acid, (□) 8-octadecene, (■) heptadecane, (♦) octadecane, (+) light HC, and (•) 8-methyl, 8-heptadecene. (---) predicted value. **(B)** The oleic and stearic acids HYD activities in function of reaction time over the MoP-S/*γ*-Al_2_O_3_-MgO catalyst. (▲) Oleic acid, (ж) stearic acid, (□) 8-octadecene, (■) heptadecane, (♦) octadecane, (+) light HC, and (•) 8-methyl, 8-heptadecene. (---) predicted value. **(C)** The oleic and stearic acids HYD activities in function of reaction time over the NiMoP-S/*γ*-Al_2_O_3_-MgO catalyst. (▲) Oleic acid, (ж) stearic acid, (□) 8-octadecene, (■) heptadecane, (♦) octadecane, (+) light HC, and (•) 8-methyl, 8-heptadecene. (---) predicted value.

The fatty acids HYD activities function of the product conversion of the NiP-S/*γ*-Al_2_O_3_-MgO, MoP-S/*γ*-Al_2_O_3_-MgO, and NiMoP-S/*γ*-Al_2_O_3_-MgO catalysts are reported after 6 h of reaction in Table 4. The catalysts did not show significant differences in the transformations of oleic and stearic acids (∼95%–99%), except in the conversion of stearic acid by NiP-S/*γ*-Al_2_O_3_-MgO (85%) as seen from Figures 7A–C, where the hydrogenation conversion of oleic acid to stearic acid was similar over the three catalysts; however, the HYD rate constant (k_3_) for catalyst NiP-S/γ-Al_2_O_3_-MgO was much lower than that for other catalysts, which must be related to the oxophilic capacity of molybdenum (Zhang et al., 2020). Hence, the effect was evaluated on the performance of the HDO, HDCx-HDCn, and HCK activities (Table 4). The pseudo-first-order rate constant values [k_1_+k_4_ (HDO), k_5_ (HDCx-HDCn) and k_6_+k_7_ (HCK)] of Scheme 1 showed that MoP-S/*γ*-Al_2_O_3_-MgO was more active at HDO, showing the highest production rate for octadecane of 53 mol/(g_cat_·h), whereas NiP-S/*γ*-Al_2_O_3_-MgO was more active at HDCx-HDCn [45 mol/(g_cat_·h)] and HCK [6 mol/(g_cat_·h)], and NiMoP-S/*γ*-Al_2_O_3_-MgO had a mix of HDO [47 mol/(g_cat_·h)] and HDCx-HDCn [41 mol/(g_cat_·h)], which showed production towards octadecane, heptadecane, and light hydrocarbons (Figures 7A–C). Likewise, MoP-S/*γ*-Al_2_O_3_-MgO and NiMoP-S/*γ*-Al_2_O_3_-MgO showed the highest HYD from oleic acid to stearic acid of 16.2 and 12.4 mol/(g_cat_·h), respectively. Therefore, the previous results confirm that MoP-S/*γ*-Al_2_O_3_-MgO and NiMoP-S/*γ*-Al_2_O_3_-MgO catalysts had a higher percentage of the HDO route (Nelson et al., 2006); while NiP-S/*γ*-Al_2_O_3_-MgO displayed a significant increase for the HDCx-HDCn and HCK routes. On the other hand, although the hydrocracking of the C18 and C17 hydrocarbons also took place in all the catalysts, their selectivity was very low on all the catalysts, considering the rate constant values (k_6_+k_7_). Although the surface area and pore size (similar) did not directly correlate with the catalytic activity, the decreasing pore volume probably allows greater exposure of the active sites to the catalysis regardless of which route is taken. In this regard, the HDO, HDCx-HDCn, and HCK activities can be correlated with the number and nature of the active sites on the surface (Lewis and Brönsted sites).

**TABLE 4:** Apparent rate constants and adsorption constants of the Al_2_O_3_-MgO supported NiMo mixed phosphidic-sulphidic phase for oleic and stearic acids hydrogenation in the reaction network shown in Scheme 1.

Catalyst	Fatty acids HYD rate constants × 10^3^ mol/(g_cat_·h)							Fatty acids HYD adsorption constants L/mol		Fatty acids HYD activities mol/(g_cat_·h)		
	k_1_	k_2_	k_3_	k_4_	k_5_	k_6_	k_7_	K_1_	K_2_	HDO	DCx-DCn	HCK
NiP-S/*γ*-Al_2_O_3_-MgO	5.1	2.3	3.7	3.6	44.7	4.5	1.8	2.51	6.46	9	45	6
MoP-S/*γ*-Al_2_O_3_-MgO	7.2	2.7	16.2	45.6	29.1	2.3	0.16	0.08	0.03	53	29	2
NiMoP-S/*γ*-Al_2_O_3_-MgO	0.31	4.0	12.4	46.7	40.9	2.4	0.036	0.25	0.45	47	41	2

HDO = k1 + k4, DCx-DCn = k5, HCK = k6 + k7.

In the case of our best catalyst, the higher HDO and HDCx-HDCn activities could be ascribed to the high hydrogenolysis capacity of Mo^δ+^/Mo^4+^ (oxophilic metal as mentioned above) on the surface combined with Brönsted and Lewis acidity of the material that preferentially increases the rate of C-O bond cleavage reactions, i.e., NiMoP-S/*γ*-Al_2_O_3_-MgO and MoP-S/*γ*-Al_2_O_3_-MgO have a lot of Brönsted acid sites compared to NiP-S/*γ*-Al_2_O_3_-MgO. Thus, the molar ratio between the HDO and HDCx-HDCn (denoted as (k_4_+k_1_)/k_5_ ratio) can represent the selectivity between the decarbonylation and HDO pathways (Table 4 and Scheme 1). It was found that the HDO/HDCx-HDCn ratios were between 0.19 and 1.81 on all catalysts at 300°C, meaning that the oleic and stearic acids were deoxygenated mainly through the HDO pathway as follows: NiP-S/*γ*-Al_2_O_3_-MgO < NiMoP-S/*γ*-Al_2_O_3_-MgO < MoP-S/*γ*-Al_2_O_3_-MgO. The metal Mo possesses a high oxophilicity; namely, Lewis acid sites on MoP-MoS_2_ [coordinatively unsaturated sites (CUS)] adsorb the oxygen favoring the activation and dissociation of C=O and C-O bonds by Brønsted acid sites, i.e., neighboring SH and/or OH groups as mentioned by Wang (Wang and Ozkan, 2005). However, NiP-S/*γ*-Al_2_O_3_-MgO, despite having many Lewis and Brønsted acid sites, showed low activity towards HDO (k_1_ vs. k_3_); this is probably due to the strength of the acid site and not to the number of Ni^δ+^ species. Chen and Ruinart reported that Ni-based catalysts such as Ni, Ni_2_P, and Ni_3_S_2_ promote the decarbonylation/decarboxylation pathway (Chen et al., 2014b; Ruinart de Brimont et al., 2012). In this regard, our NiP-S/*γ*-Al_2_O_3_-MgO catalyst exhibited HDCx-HDCn as the predominant route, suggesting that HDCx-HDCn could be related to the absence of sulfides; which is consistent with our results of XPS and Raman for NiP-S/γ-Al_2_O_3_-MgO that does not present an Ni sulfide on the surface. While the XPS and Raman results of MoP-S/γ-Al_2_O_3_-MgO and NiMoP-S/γ-Al_2_O_3_-MgO showed the presence of sulfides. Thus, a synergistic effect between MoS2 on the surface and the phosphide (phosphidic-sulphidic composite) would be an accurate representation for the active phase in typical hydrotreating conditions in these materials (Nelson et al., 2006). Hence, even though the selectivity behavior is completely the same between the phosphides and conventional sulfidic catalysts for oleic and stearic acids (Kaluža et al., 2019), this result agrees well with the previous report by Shim et al. (2015), showing the possible deoxygenation reaction pathways of oleic acid. Similarly, Coumans and Hensen, (2017) have suggested that in the NiMo sulfide the catalyst surface contains different sites for direct HDO and DCx-DCn reactions. On the side of the phosphides and carbides, Ni phosphides tend to follow the route of DCx and DCn with excellent conversions of fatty acids (Zhang et al., 2016; Ruangudomsakul et al., 2021); but reflects an intrinsic activity significantly lower than those obtained in this research; whilst Yun et al. (2017) reported that for NiMoP catalysts, Ni sites governed catalytic activity and Mo sites controlled the selectivity; however, our study shows that Ni tends to the route of DCx and DCn, and Mo the HDO. This is possibly because the phosphidic-sulphidic mixed-phase samples may have different active sites, which can improve DCx-DCn and HDO performance of catalysts.

To sum up, the catalytic activity obtained in HDO, HDC, and HCK may be correlated with the presence at the surface of Mo and Ni species, hence Mo^δ+^-Mo^4+^ in MoP-S/*γ*-Al_2_O_3_-MgO and Ni^δ+^ in NiP-S/*γ*-Al_2_O_3_-MgO favors HDO and DCx-DCn, respectively; hence the combination of Mo^δ+^-Mo^4+^ and Ni^δ+^ species in NiMoP-S/*γ*-Al_2_O_3_-MgO increases HDO and DCx-DCn; while for HCK, the Ni species have the most significant effect because MoP-S/*γ*-Al_2_O_3_-MgO and NiMoP-S/*γ*-Al_2_O_3_-MgO are less active towards HCK.

Determining the adsorption constants for the oleic and stearic acids HYD in the proposed mechanism could help us understand the different activities between the NiP-S/*γ*-Al_2_O_3_-MgO, MoP-S/*γ*-Al_2_O_3_-MgO, and NiMoP-S/*γ*-Al_2_O_3_-MgO catalysts. The data correlation is shown in Figures 7A–C, comparing experimental versus predicted yield. We found in the kinetic analysis that using the rate expressions Z = 2 for the inhibition terms gave better fitting to the data. Differences may rationalize this among reactions taking place in the HYD network of fatty acids, and based on our results, this leads us to think that there are two types of “bifunctional” sites (Infantes-Molina et al., 2015; Lee and Oyama, 2006). Therefore, we could designate, according to Scheme 1, a site (S1) for oleic acids and a site (S2) for stearic acids, where the hydrogenation (HYD) and HYG can occur in either of these two sites (BRIM and CUS sites, i.e., Lewis and Brønsted acid sites). At site 1, the oleic acid can be hydrogenated and hydrodeoxygenated to stearic acid and 8-octadecene, and the latter is subsequently isomerized to 8-methyl, 8-heptadecene. At the same time, site 2 stearic acid can be hydrodecarboxylated and hydrodeoxygenation to heptadecane and octadecane, which are hydrogenated to form low molecular weight compounds called here light hydrocarbons. In addition to this type of bifunctional site, the acid strength and number give preponderance to the proportion generated for each reaction route. Hence, the adsorption constants increase, for all the catalysts, in the following order: MoP-S/*γ*-Al_2_O_3_-MgO < NiMoP-S/*γ*-Al_2_O_3_-MgO < NiP-S/*γ*-Al_2_O_3_-MgO.

The difference between the adsorption constants found applying this model suggests that the strength and number of Lewis sites in NiP-S/γ-Al2O3-MgO cause a high adsorption strength of the fatty acids on the catalyst surface (XPS results do not show sulfur species); otherwise it is observed in MoP-S/γ-Al_2_O_3_-MgO and NiMoP-S/γ-Al_2_O_3_-MgO. It seems that there is a type of synergism between the Lewis and Brönsted sites of the catalyst that achieves an optimal strength of the bond between the relevant intermediate and the catalyst as exposed in the classical Sabatier principle, which states that there is an optimum “bond strength” defining the best catalyst for a given reaction (Medford et al., 2015). We consider that phosphide acts as a structural promoter in the catalysts with sulfide species on the surface and a kind of synergism is present in the phosphidic-sulphidic mixed-phase, allowing the largest generation of heptadecane and octadecane by BRIM sites for HYD and CUS sites for HYG.

## Conclusion

The effect of phosphidic-sulphidic phase content in MoP-S/*γ*-Al_2_O_3_-MgO, NiMoP-S/*γ*-Al_2_O_3_-MgO, and NiP-S/*γ*-Al_2_O_3_-MgO on hydrogenation of fatty acids was evaluated. The chemical analyses by FRX for MoP-S/*γ*-Al_2_O_3_-MgO, NiMoP-S/*γ*-Al_2_O_3_-MgO, and NiP-S/*γ*-Al_2_O_3_-MgO showed stoichiometric values for Al:Mg, Ni:Mo, Ni:P, and Mo:Ni:P of 38 and ∼1, while EDS spectra and elemental mapping confirmed the presence of Mo, Ni, Al, O, P, Mg, and S with good distribution on Al_2_O_3_-MgO. The XRD patterns revealed differences between metallic phosphates and the phosphidic-sulphidic phase. However, it was difficult to distinguish a phosphide phase because the crystallite size was too small, and the phosphide particles were well dispersed on the support. MoP-S/*γ*-Al_2_O_3_-MgO and NiMoP-S/*γ*-Al_2_O_3_-MgO showed two typical Raman peaks at 380 and 405 cm^−1^ for the E^1^_2g_ and A_1g_ active modes characteristic of the bulk of multilayered MoS_2_. All the N_2_ physisorption isotherms of the materials were type IV with H1 and H2 hysteresis loops. MoP-S/*γ*-Al_2_O_3_-MgO and NiP-S/*γ*-Al_2_O_3_-MgO showed better textural properties than NiMoP-S/*γ*-Al_2_O_3_-MgO with an average pore radius between 6.5 and 11.2 nm. XPS evidenced Mo^δ+^(0 < δ+ < 2), Mo^4+^, Mo^6+^, Ni^δ+^(0 < δ+ < 2), Ni^2+^, P^δ+^, and P^5+^ species, which showed a dependence with the type of metal and the presence of sulfur. The FTIR Py analysis of MoP-S/*γ*-Al_2_O_3_-MgO, NiMoP-S/*γ*-Al_2_O_3_-MgO, and NiP-S/*γ*-Al_2_O_3_-MgO showed the main band characteristics of the pyridine adsorbed on Lewis acid sites and much lower amounts of Brønsted acid sites. The NiMoP-S/γ-Al_2_O_3_-MgO catalyst had a more significant number of Brønsted and Lewis sites (15.7–34.2 μmol/g vs. 36.9–106.6 μmol/g). However, the Lewis sites in NiP-S/γ-Al_2_O_3_-MgO were stronger that NiMoP-S/γ-Al_2_O_3_-MgO. MoP-S/*γ*-Al_2_O_3_-MgO was more active at HDO showing the highest production rate for octadecane of 53 mol/(g_cat_·h), whereas NiP-S/*γ*-Al_2_O_3_-MgO was more active at HDCx-HDCn [45 mol/(g_cat_·h)] and HCK [6 mol/(g_cat_·h)], and NiMoP-S/*γ*-Al_2_O_3_-MgO had a mix of HDO [47 mol/(g_cat_·h)] and HDCx-HDCn [41 mol/(g_cat_·h)]. The higher HDO and HDCx-HDCn activities could be ascribed to the high hydrogenolysis capacity of molybdenum sulfide (oxophilic metal). The (k_4_+k_1_)/k_5_ ratios were between 0.19 and 1.81 on all catalysts at 300°C, meaning that the oleic and stearic acids were deoxygenated mainly through the HDO pathway as follows: NiP-S/*γ*-Al_2_O_3_-MgO < NiMoP-S/*γ*-Al_2_O_3_-MgO < MoP-S/*γ*-Al_2_O_3_-MgO. The catalysts had bifunctional sites, leading to an increase of the adsorption constants as follows: MoP-S/*γ*-Al_2_O_3_-MgO (K_1_ = 0.08 and K_2_ = 0.03 L/mol) < NiMoP-S/*γ*-Al_2_O_3_-MgO (K_1_ = 0.25 and K_2_ = 0.45 L/mol) < NiP-S/*γ*-Al_2_O_3_-MgO (K_1_ = 2.5 and K_2_ = 6.5 L/mol). Hence the high production of heptadecane and octadecane by the presence of mixed NiMo phosphidic-sulphidic species (BRIM sites for HYD and CUS sites for HYG) suggests a type of synergism between the Lewis and Brönsted sites of the catalyst giving an optimum “bond strength” defining the best catalyst for a given reaction.

## Data Availability

The original contributions presented in the study are included in the article/Supplementary Material, further inquiries can be directed to the corresponding author.
